# Non-adherence to Clinical Practice Guidelines in Regional Anesthesia and Pain Interventions: Insights From a Comprehensive Case Series Review

**DOI:** 10.7759/cureus.75847

**Published:** 2024-12-17

**Authors:** Salah N EL-Tallawy, Joseph V Pergolizzi, Marium J Albasher, Nawwaf S Alghamdi, Gehan I Salem, Rania S Ahmed, Abdullah M KaKi, Ahmed K Thallaj, Ali H Alwahabi, Amro M Amer, Radwa H Ahmed

**Affiliations:** 1 Anesthesia and Pain Management, College of Medicine, King Khalid University Hospital, King Saud University, Riyadh, SAU; 2 Anesthesia and Pain Management, Faculty of Medicine, Minia University and NCI, Cairo University, Cairo, EGY; 3 Pain Management, NEMA Research Inc., Naples, USA; 4 Anesthesiology, King Khalid University Hospital, King Saud University, Riyadh, SAU; 5 Rheumatology and Rehabilitation Department, Faculty of Medicine, Assiut University, Assiut, EGY; 6 Family Medicine and Polyclinics, Alfaisal University College of Medicine, Riyadh, SAU; 7 Pain Medicine, Alsalama Hospital, Jeddah, SAU; 8 Pain Medicine, Faculty of Medicine, King Abdulaziz University, Jeddah, SAU; 9 Anesthesia, College on Medicine, King Saud University, Riyadh, SAU; 10 Neurophysiology, King Khalid University Hospital, King Saud University, Riyadh, SAU; 11 Clinical Pathology Department, Faculty of Medicine, Assiut University, Assiut, EGY

**Keywords:** epidural lipomatosis, imaging tools, intercristal line, neuraxial analgesia, neurological complications, neurophysiological studies, peripheral nerve block, ultrasound-guided nerve block

## Abstract

Adhering to established guidelines, regional anesthesia (RA) and pain interventions are essential for preventing or minimizing the risk of complications. This study examines neurological complications that may arise when RA or pain interventions are performed without adherence to the clinical practice guidelines. This article aimed to emphasize the relationship between deviations from standards of care in RA and neurological outcomes. Specifically, it focuses on neurological deficits associated with RA and pain interventions. This retrospective study analyzed five cases, selected from 18 cases meeting the inclusion criteria, reviewed over a period from 2012 to 2023. The data collected included patient demographics, anesthesia details, neurological deficits, diagnostic findings, management approaches, and outcomes at three- to six-month follow-ups. The article presents five cases, each examining the techniques used, clinical diagnosis, radiological imaging, neurophysiological studies, and the management protocols involved. Furthermore, it highlights the consequences of deviations from the standard of care and their impacts on neurological outcomes. Outcomes and key lessons learned from each case are discussed. Five cases were analyzed, revealing deviations from the standard of care such as incorrect spinal needle placement, injection despite warning signs, inadequate patient evaluation, and procedures performed by inexperienced anesthetists. While some patients achieved full recovery, others experienced lasting deficits. The study emphasizes the critical importance of strict adherence to clinical practice guidelines in RA and pain interventions to minimize neurological complications. It highlights the essential roles of proper training, precise technique, thorough patient evaluation, patient-centered care, informed consent, and the use of advanced diagnostic tools to mitigate risks and improve patient safety and outcomes.

## Introduction

The incidence of neural injury in regional anesthesia is higher than previously anticipated [1], with regional anesthesia accounting for approximately one-fifth of professional liability claims [2]. While most complications associated with regional anesthesia and pain interventions are minor and transient, typically resolved within a few days, permanent or persistent neurologic complications remain rare. However, even transient complications can create high levels of anxiety for patients, anesthetists or pain physicians, and family members [3,4]. Data suggest that the incidence of transient complications is less than 1%, while permanent neurological deficits occur in approximately 0.01% to 0.03% of cases [2,3].

Strategies to reduce neurological complications following regional anesthesia emphasize adequate training and strict adherence to clinical practice guidelines. Key measures include maintaining an aseptic technique [1,2], using appropriate doses and concentrations of local anesthetic, employing imaging techniques when necessary, and avoiding injection when red flags, such as pain or high resistance during injection are present [2,3]. Training plays a critical role in preventing neurological complications after regional anesthesia, as it enhances both practitioner skills and ensures patient safety [3,5]. In addition, the use of the WHO Safe Surgical Checklist is essential for ensuring proper patient identification and verification of the surgical site [6].

This article highlights the risks associated with deviations from the safety guidelines through a series of cases that illustrate the clinical implications of such deviations. It explores the underlying mechanisms, diagnostic tools, management approaches, and outcomes related to these cases. Furthermore, the article provides clinical guidelines and recommendations to prevent or minimize the neurological injury resulting from regional anesthesia and pain interventions.

## Case presentation

Study design

A retrospective review was conducted for patients at King Khalid University Hospital, King Saud University, Saudi Arabia, covering the period from 2012 to 2023. The data of this study was part of a quality improvement program aimed at improving postoperative pain outcomes. The study was approved by the Institutional Review Board (IRB) of the College of Medicine, King Saud University, Riyadh, Saudi Arabia (Ref. No. 18/0443/IRB). 

Eighteen cases with neurological symptoms following regional anesthesia and pain interventions were initially identified. Of those, five patients meeting the inclusion criteria were included in this case series analysis. All selected cases experienced neurological deficits after regional anesthesia or pain interventions, with four cases occurring post-regional anesthesia and one case following a pain intervention. Patients were identified by the Acute Pain Service (APS), the primary anesthesia team, or referred to pain management by the primary surgical team.

Inclusion and Exclusion Criteria

Inclusion criteria encompassed patients with documented neurological deficits following regional anesthesia or pain interventions. Patients identified within 24 hours post-procedure, including in-patients. Exclusion criteria included patients with complications attributable to surgical procedures, transient neurological complications resolving within a few hours, those lacking adequate documentation, uncomplicated cases of post-dural puncture headache (PDPH), and patients with pre-existing neurological deficits prior to the planned procedure.

Data Collection

Demographic characteristics including age, gender, and medical history, were collected alongside details of the surgical procedure, anesthetic technique, and the types of regional anesthesia or pain intervention performed. Specific details of the neurological deficits such as onset, symptoms, signs, course, and associated deficits, were documented. Additionally, findings from diagnostic tools such as X-ray, CT, MRI, and neurophysiological studies were recorded. The multidisciplinary management provided was also recorded. Finally, the pathophysiological type and diagnosis of the neurological injuries were categorized, and deviations from the standard of care were identified. Outcomes at long-term follow-up (three to six months) and key lessons learned from the case scenarios were documented.

Individual case analysis

First Case

Preoperative assessments: A 48-year-old male patient with a history of hypertension and obesity (BMI = 39 kg/m^2^) underwent preoperative evaluation. Laboratory tests were within normal limits, and no additional medical issues were identified. The patient was classified as ASA II according to the American Society of Anesthesiologists physical status classification. He was scheduled for bilateral open inguinal hernia repair under combined spinal-epidural anesthesia (CSE). After completing the preoperative assessment, informed written anesthesia consent was obtained, and premedication was administered.

Anesthetic technique: The patient was positioned sitting, and his back was sterilized. Local infiltration of 3 ml of 1% lidocaine was administered at the selected intervertebral space. An epidural needle was inserted at the L2-L3 intervertebral space, and the epidural space was confirmed using the loss-of-résistance technique. A 27-G spinal needle was then inserted through the epidural needle, resulting in cerebrospinal fluid (CSF) flow. During the spinal needle insertion, the patient experienced severe, intolerable, shouting, and electric-like pain radiating down the right lower limb and right foot. To alleviate the pain, the anesthesia team immediately administered a bolus dose of heavy bupivacaine (2 ml of 0.5%) intrathecally. The spinal needle was then removed, and an epidural catheter was inserted and secured at a depth of 5 cm inside the epidural space. During the surgery, three boluses of bupivacaine (10 ml of 0.25% each) were administered through the epidural catheter after confirming negative aspiration for blood or CSF. Throughout the three-hour procedure, the patient received heavy sedation with midazolam (5 mg IV) and a propofol infusion (100 mcg/kg/min). Additional intravenous analgesia included paracetamol (1 g IV), lornoxicam (16 mg IV), and fentanyl (150 mcg in divided IV boluses) to manage intraoperative pain.

Postoperative care: At the conclusion of surgery, the patient was transferred to the post-anesthesia care unit (PACU), where the primary anesthesia team initiated a continuous infusion of bupivacaine (5 ml/hour of 0.0625% combined with 2 mcg/ml fentanyl). After 30 minutes, the patient was safely transferred to the ward. 

The event: The APS was notified at 9:00 PM on the same day of surgery due to persistent bilateral motor weakness and numbness in both lower limbs. These symptoms were disproportionate to the local anesthetic infusion dose and concentrations. An immediate examination by the APS resident revealed a heavy motor block in both lower limbs, more prominent on the right side, accompanied by paresthesia and neuropathic pain in the right lower limb and the perineal region. There was no back tenderness, signs of meningeal irritations, or other abnormal findings.

Management protocol: The local anesthetic infusion was stopped immediately, and patient-controlled analgesia (PCA) with fentanyl was initiated for acute postoperative pain management. The APS re-evaluation on the following postoperative day showed bilateral asymmetrical motor weakness and hypoesthesia, particularly affecting the right lower limb and perineal region. The patient experienced neuropathic pain in the right lower limb and perineal region, with significant discomfort experienced in the right foot. There were no red flags such as localized back tenderness, neck rigidity, fever, nausea, or vomiting.

The primary anesthesia team and surgical team were informed, and further investigations were ordered, including an urgent MRI of the lumbosacral spine and neurophysiological studies (electromyography (EMG) and nerve conduction studies (NCS)). Neurology and physical therapy consultations were also requested. For epidural catheter removal, heparin was held for 12 hours, and the laboratory tests confirmed a normal coagulation profile. The epidural catheter was removed safely, and postoperative pain management continued with PCA fentanyl supplemented by Gabapentin (400 mg orally every eight hours) for neuropathic pain.

By postoperative day two, significant improvement in motor weakness and sensory deficits was observed in the left lower limb. In comparison, only minimal improvement was noted in the right lower limb exhibited at the hip and Knee joints, with no improvement at the right foot. Both acute postoperative pain and neuropathic pain were significantly alleviated by medical treatment.

**Figure 1:**
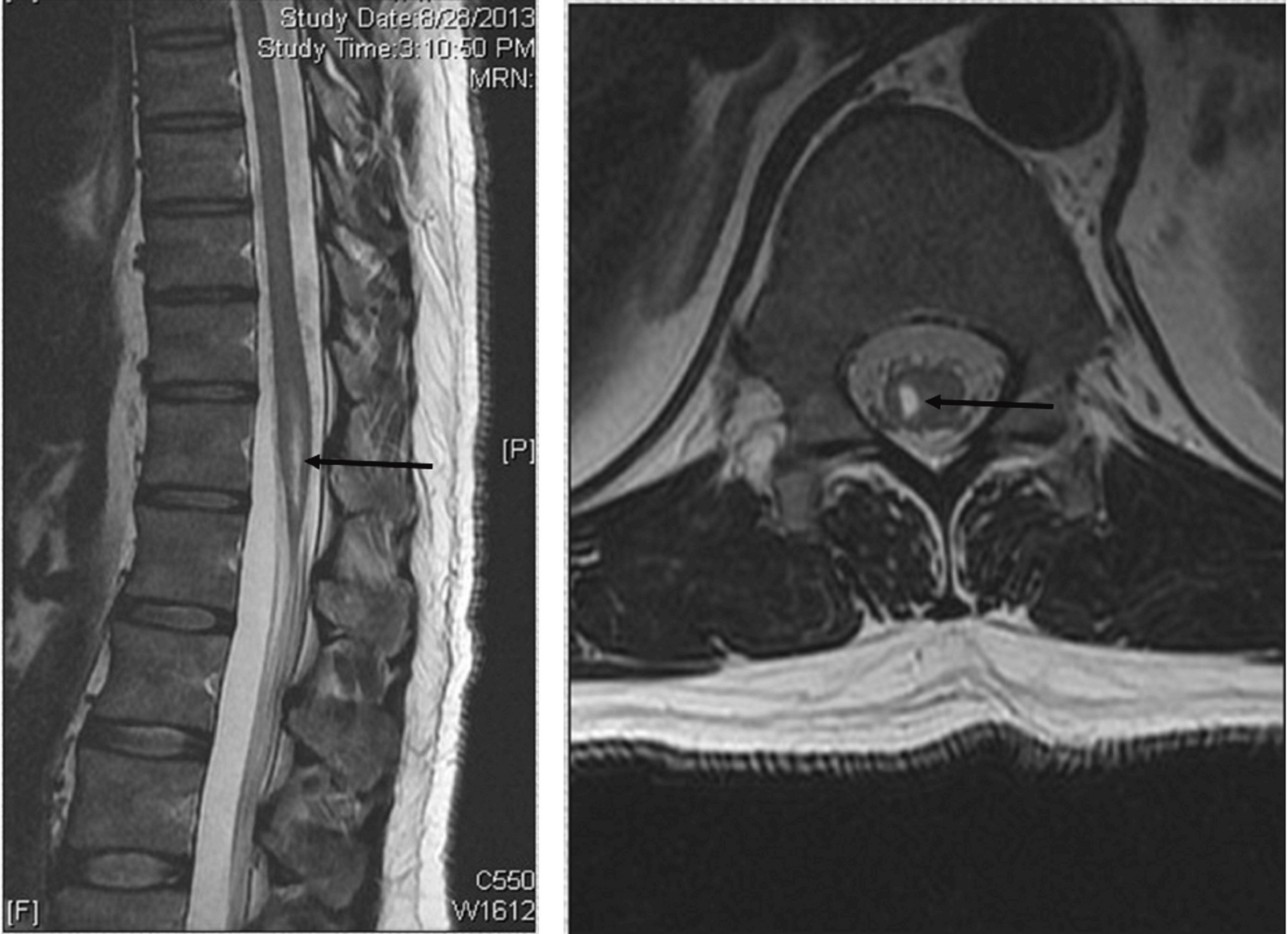
MRI: Sagittal T2-weighted (left) and axial T2-weighted (right) images of the lumbosacral spine demonstrate a lesion in the spinal cord at the level of the conus medullaris, extending from the L1 to L2 levels.

MRI results revealed clear evidence of spinal cord injury at the level of the conus medullaris (Figure 1). Neurophysiological studies showed no injury to the peripheral nerves of either side (e.g., peroneal and tibial nerves) as shown by NCS. EMG showed poor muscle activation in the tibialis anterior (TA), extensor hallucis longus (EHL), tibialis posterior (TP), and gastrocnemius muscles bilaterally.

Neurology consultation: The neurological examination and the proposed treatment plan were consistent with those recommended by the APS. The initial diagnosis by the neurology team was anesthesia-induced radiculopathy versus plexopathy. Continuous follow-up demonstrated progressive improvement in motor power and sensation, with complete recovery of the left lower limb by postoperative day four. The right side showed gradual improvement at the hip and knee, though at a slower rate than the left side.

Upon discharge after 10 days, the motor power of the right foot showed plantar flexion at 4+/5 and dorsiflexion at 2+/5. The patient was able to walk unsupported with a slight limp (high-stepping gait) on the right side due to foot drop, as well as residual hypoesthesia and neuropathic pain around the perineal area, inner side of the right thigh, knee, lateral aspect of the leg and the foot.

Final outcome: At the six-month follow-up, the patient still exhibited foot drop (with right foot plantar flexion at 4+/5 and dorsiflexion at 3/5) and persistent neuropathic pain in the right lower limb.

Second Case

Preoperative assessments: A 75-year-old female patient was scheduled for a left total knee replacement (TKR). The preoperative assessment revealed comorbidities, including hypertension, diabetes, and dyslipidemia, all managed and within the acceptable ranges.

Anesthetic technique: The patient received combined spinal-epidural anesthesia (CSE) for left TKR with light sedation, in line with standard practices for regional anesthesia. The anesthesia and surgery were conducted smoothly and uneventfully, lasting two and a half hours.

Postoperative care: At the conclusion of surgery, the patient was transferred to the PACU. Postoperative analgesia was initiated in the PACU by the primary anesthesia team with continuous epidural analgesia through an epidural catheter, delivering a local anesthetic infusion at a rate of 5 ml/hour of 0.0625% bupivacaine combined with 2 mcg fentanyl/ml. After 30 minutes, the patient was safely transferred to the ward.

The event: The following morning, and during the routine APS round, an examination of the patient revealed an inability to dorsiflex the left foot (foot drop) on the same side of surgery. In addition, there was hyposthesia noted along the lateral aspect of the left foot, with no other deficits or red flags. The right lower limb was completely normal.

Management protocol: The APS team immediately stopped the local anesthetic infusion and prescribed PCA fentanyl for postoperative pain management. Following protocols for neurological injury after neuraxial anesthesia, the APS team ordered consultations with neurology, physical therapy, and rehabilitation consultations. An urgent MRI for the lumbosacral spine and neurophysiological studies (EMG and NCS) were also requested. To prepare for epidural catheter removal, heparin was stopped, and a coagulation profile and CBC were ordered. The epidural catheter was safely removed after 12 hours. Both the primary orthopedic and anesthesia teams were notified.

A multidisciplinary team meeting was convened, including the orthopedic, primary anesthesia, and APS teams. The orthopedic team verified the same deficits, as described by the APS, and diagnosed the injury as limited to the left common peroneal nerve. The peroneal nerve, a branch of the sciatic nerve, is responsible for motor and sensory functions in the lower leg and foot. Its injury can lead to foot drop, sensory loss, or weakness. The orthopedic team diagnosed the neurological deficit as common peroneal nerve neuropraxia, a recognized and transient complication following TKR. In addition, they confirmed that this complication, occurring at the same site of surgery, was unrelated to the anesthetic technique. Consequently, the orthopedic consultant canceled the investigations and initiated their treatment plan including the removal of all tight dressings to relieve potential nerve compression and repositioning of the knee to alleviate nerve tension. They also ruled out other possible causes, such as postoperative hematoma or direct nerve injury.

Final outcome: The patient showed significant improvement over the next few weeks, achieving full recovery within 3 months of the procedure.

Third Case

Preoperative assessments: A 22-year-old female patient, with no significant medical history, was scheduled for minor orthopedic surgery below the right knee.

Anesthetic technique: The patient received general anesthesia with a laryngeal mask airway. While the patient was under general anesthesia, an anesthesia resident performed an ultrasound-guided (USG) sciatic-popliteal nerve block using 15 ml of 0.25% bupivacaine. The intraoperative course was uneventful since the procedure was conducted under general anesthesia.

Postoperative care: At the conclusion of the surgery, the patient was safely transferred to the PACU for 30 minutes, before being moved to the ward.

The event: The following morning, the orthopedic team contacted the APS and the primary anesthesia team regarding a delayed recovery from the sciatic nerve block. The patient reported numbness and heaviness in the right leg, with right foot drop and neuropathic pain on the same side of the surgery. Neurological evaluation for the motor power revealed dorsiflexion at 1/5 and plantar flexion at 3/5.

Management protocol: Both the APS and the orthopedic teams requested consultations with neurology, physical therapy, and rehabilitation. They also ordered a neurophysiological study (EMG and NCS) and an urgent MRI of the right knee (operative site). The MRI results revealed no abnormal findings at the surgical site, such as compression or hematoma. Meanwhile, the neurophysiological studies confirmed nerve injury, with the lesion located above the level of the surgery. The neurology consultant confirmed the diagnosis of regional nerve block-induced nerve injury. The patient was enrolled in a physiotherapy and rehabilitation program. She experienced slight improvement during the following few days after surgery and was discharged after five days.

An evaluation of the patient before discharge showed residual motor weakness in the right foot, with dorsiflexion at 2+/5 and plantar flexion at 4/5. The patient was continued follow-up with the neurology, physical therapy, and pain management clinics. She was prescribed medications for neuropathic pain, including pregabalin (75 mg PO/BID) and amitriptyline (10 mg daily). At a three-month follow-up, she still exhibited motor weakness in the right foot, presenting by dorsiflexion at 3/5, planter flexion at 4/5, along with neuropathic pain involving the foot and the lateral aspect of the leg.

Final outcome: After three months, the patient showed some improvement in the right foot drop, with dorsiflexion at 3+/5, and planter flexion at 4+/5, although neuropathic pain persisted in the foot and the lateral aspect of the leg.

Fourth Case

Preoperative assessment: A 38-year-old female teacher, a divorced single mother of two children, presented to the pain clinic complaining of chronic low back pain (LBP). She was on multiple medications and pain relievers. She was obese (BMI 38 kg/m^2^) with no other medical or surgical issues. An MRI of the lumber spine, performed two years ago, revealed multilevel degenerative disc disease, more pronounced at the L4-5 and L5-S1 levels. The pre-procedure evaluation revealed no motor or sensory deficits, and her sphincters were intact.

Pain intervention: The patient was scheduled for a fluoroscopic-guided lumbar epidural steroid injection (ESI) to relieve her LBP. She was positioned prone with a pillow under the pelvis to straighten the lumbar lordosis. After sterilization and local skin infiltration by 3 ml of 1% lidocaine, an epidural needle was inserted at the L4-L5 level. The epidural space was confirmed by the loss of resistance and the characteristic distribution of the contrast medium. After negative aspiration for blood and CSF, a total volume of 6 mL was injected, consisting of 2 ml of 80 mg Depo-Medrol and 4 ml of 0.25% bupivacaine.

Post-procedure care: The procedure was uneventful, and after 30 minutes, the patient was transferred safely to the daycare surgery unit.

The event: Two hours later, at 4:00 PM, a routine evaluation of the patient before discharge revealed bilateral lower limb weakness and hyposthesia. A detailed neurological examination identified patchy hypoesthesia in both lower limbs. Motor power assessment showed right hip, knee, and foot ranged from -3/5 to +3/5, while the motor power in the left foot was 2/5, and in the left knee and hip was 1/5. Sphincters were intact, with no signs of meningeal irritation or other red flags.

**Figure 2:**
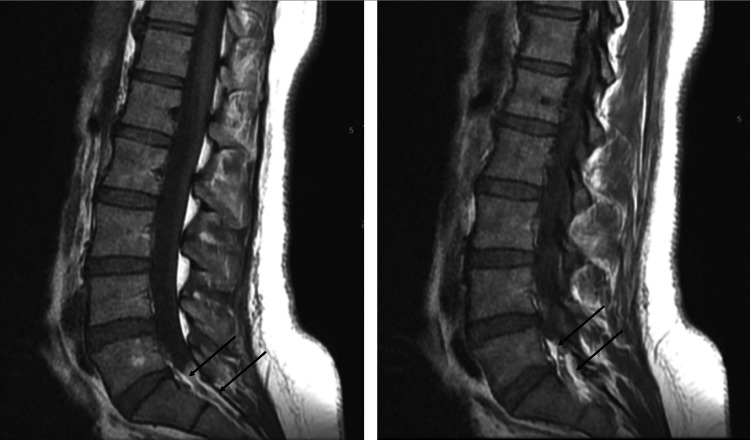
MRI (sagittal T1-weighted) of the lumbosacral spine revealed thecal sac narrowing at the L5 level, with complete effacement of the thecal sac at the S1 and S2 levels. These findings are consistent with epidural lipomatosis following a steroid injection.

Management protocol: An urgent MRI scan was performed at 7:00 PM, confirming multilevel degenerative disc disease, most notable at L4-5 and L5-S1, without significant neural canal compressive lesions (e.g., no epidural hematoma or abscess). However, there was interval development of the thecal sac narrowing at the lower L4 and L5 levels with almost complete effacement of the thecal sac at S1 and S2 levels. High T1 and T2 signal intensities were also noted in the epidural space of the sacral canal suggesting possible epidural lipomatosis following the previous epidural steroid injections (Figure 2).

Neurosurgery consultation: A neurosurgical consultation was conducted the same day (at 9 PM). The right lower limb showed significant improvement (4+/5 in all muscle groups), while the left lower limb remained unchanged (left foot 2/5, knee and hip 1/5). Deep reflexes (ankle and knee) were preserved the sphincters were intact. Sensation in the left lower limb was diminished, accompanied by mild to moderate LBP. There was no role for the surgical intervention. Pain management with (gabapentin 400 mg PO twice daily) and dexamethasone (8 mg IV every eight hours) was initiated, with follow-up planned.

Neuropsychiatry consultation: On the following day, a neuropsychiatric consultation was conducted. The patient reported moderate to severe LBP radiating to the left foot and up to the left buttock, exacerbated by movement of the left lower limb. Examination revealed intact motor power in the upper limb (5/5 all over), normal muscle tone bilaterally, and intact sensation. In the left lower limb, motor power was assessed as follows: hip flexion 4/5 (with back pain on movement), hip abduction 3/5, hip adduction 4/5, knee flexion 3/5, knee extension 2/5, ankle planter and dorsiflexion 3/5, ankle eversion and inversion 3/5. Muscle tone was normal bilaterally. Sensory examination showed decreased pinprick sensation in L5, S1, and S2 dermatomes of the left lower limb compared to the right. The patient was able to walk only with assistance. Examination of the right lower limb showed intact both sensory and motor systems.

Follow-up neuropsychiatry consultation: Three days later, a follow-up neuropsychiatry consultation showed the patient still complained of LBP radiating to the left lower limb but was able to ambulate with assistance for 30 minutes in the morning. Motor examination of the left lower limb showed some improvements, foot dorsiflexion 5/5 and plantar flexion 4/5. Left knee flexion 4/5 and knee extension 3/5. Left hip flexion could not be assessed due to the associated pain and the patient not giving full power. Weakness was likely to be secondary to severe pain; however, acute neuropathic injuries could not be totally excluded.

Neurophysiology studies: NCS and EMG were conducted two days after the incidence and showed normal study with no electrophysiological evidence of neuropathic injuries in the lower limbs. However, normal NCS and EMG can be expected in the early stages of neuropathic injuries.

Follow-up advanced neurophysiological studies: Five days after the epidural injection, advanced neurophysiological tests including somatosensory evoked potentials (SSEP) for the upper and lower limbs, and trans-cranial motor evoked potentials (MEP) were performed under sedation with propofol infusion in the operative room. No muscle relaxant was given. Tongue bite blocks were inserted for safety issues. Bilateral ulnar and posterior tibial nerve (PTN) SSER cortical waves were monitorable, symmetrical, and exhibited normal latency and amplitude values. Bilateral L4-S1 MEP compound muscle action potentials were recordable and with no latency or amplitude changes compared to the normal adult female population in either the tibialis anterior, medial gastrocnemius or abductor hallucis muscles. The Patient’s normal PTN results indicated normal functioning of proprioception sensation in the lower limbs, while lower limbs MEP findings below L4 level imply normal power in bilateral leg and foot muscles without conduction block or neuropathy (weakness) in alpha motor neurons at level L4, L5, and S1. No weakness or neuropathy were detected from conducted MEP tests. Although spinal canal stenosis due to epidural lipomatosis cannot be completely excluded, other potential causes including local anesthetic neurotoxicity and psychological reasons need to be ruled out.

Physical therapy: The physical therapy and rehabilitation consultations were clearly explained to the patient. She began a physical therapy course for two weeks. During that time, the physiotherapy team noted from the patient’s old records that she had developed similar symptoms of lower limb weakness following a previous L-ESI at another hospital two years ago and she fully recovered within two weeks. Unfortunately, the patient did not disclose this history and denied receiving L-ESI injections twice before. The first injection was at our center three years ago, and it was uneventful. The second injection was performed at another facility two years ago, and the later injection was followed by transient motor weakness for two weeks.

Final outcome: The patient was discharged after one week and she continued follow-up with the pain clinic neuropsychiatry and physical therapy. Although she continued to experience mild to moderate low back pain during movements, she achieved full recovery within four weeks after the procedure.

Fifth Case

Preoperative assessments: A 36-year-old female single with advanced rheumatoid arthritis, which makes endotracheal intubation difficult. She has been followed in the rheumatology clinic. The patient was also under psychiatric care but was noncompliant with both medications and regular visits to the psychiatrist. She was taking multiple medications including immune-suppressants, corticosteroids, NSAIDs, and antidepressants.

Anesthetic technique: The patient was scheduled for a right hip arthroplasty (THA) due to developmental dysplasia of the hip (DDH). The anesthesia plan included balanced anesthesia (general anesthesia with continuous epidural analgesia) for perioperative pain management. Initially, the patient signed the consent for general anesthesia but declined the back needle (epidural anesthesia). However, after the team explained its benefits, especially for postoperative pain control and rehabilitation, she gave oral consent only. The epidural technique was performed while the patient was in the lateral decubitus position under general anesthesia.

Postoperative care: Following a four-hour procedure, the patient was shifted to the PACU for 30 minutes before being transferred to the ward. Postoperative pain was managed with continuous epidural analgesia. The local anesthetic infusion (5 ml/hour of bupivacaine 0.0625% combined with 2 mcg/ml fentanyl) was initiated by the primary anesthesia team in the PACU.

The event: During the routine daily round of the APS the next morning, the patient was found to have bilateral lower limb weakness and hypoesthesia, which is not expected to persist more than one to two hours after using a low volume of very diluted local anesthetic (5 ml of bupivacaine 0.0625%). 

Management protocol: The continuous epidural local anesthetics infusion was immediately discontinued. An urgent MRI of the lumbosacral spine, neuropsychiatry consultation, and neurophysiological studies were ordered. For neuropathic pain, gabapentin 400 mg every eight hours was described, along with paracetamol 1 gm IV every six hours and lornoxicam 8 mg IV every eight hours as a part of the multimodal analgesia plan for postoperative pain.

MRI results: The MRI showed normal findings, with no evidence of spinal cord or nerve compression being identified.

Neurophysiological studies: The findings revealed reduced motor unit action potential amplitude and voluntary contraction, indicating weakness of motor units. Weakness and slowed nerve conduction velocities were noted along the affected nerve roots, accompanied by reduced sensory nerve action potential amplitudes and delayed transmission times. Based on these findings, local anesthetic neurotoxicity could not be ruled out.

Neurological examinations: The examination revealed bilateral lower limb weakness involving the hip, knee, and ankle joints, more pronounced in the proximal muscles, rendering the patient unable to move. Deep reflexes were diminished or absent with an asymmetrical distribution in both lower limbs, and there was reduced muscle tone in the affected areas. Sensory examination revealed hyposthesia, and numbness, with areas of paresthesia, hyperesthesia, and neuropathic pain according to the involved nerves in an asymmetrical distribution. Sphincter control remained intact. These findings suggested a lower motor neuron injury.

Neuropsychiatry consultation: The patient was found to be extremely anxious and worried about her physical condition. She was complaining of insomnia and fear of permanent disability. The team added amitriptyline 25 mg orally once daily along with psychological support and behavioral therapy.

Physiotherapy and rehabilitation: The patient began a physiotherapy and rehabilitation program. Regular follow-ups with multidisciplinary team management including the APS team, neuropsychiatry, physical therapy, and rehabilitation revealed significant improvement during the following weeks until near-complete recovery within three months.

Final outcome: Full recovery was achieved after three months of the initial event.

## Discussion

Two essential conditions for a successful regional block are accurate needle placement (e.g., correct puncture of the dura mater) and precise injection of the local anesthetic agents at the target site (e.g., intrathecal injection) [7,8]. When a block fails or leads to complications, it is usually attributed to one of three aspects: technical failure, lack of experience, or non-adherence to clinical practice guidelines. These basic principles are fundamental for the success of any regional anesthesia or pain intervention, and deviations from these standards are often the primary causes of failure or complications [8,9]. This case series highlights deviations from these core principles, which are discussed in the following comprehensive analysis.

First case

Spinal cord injury highlights two major deviations from standard care practices: incorrect spinal needle placement due to neglect of anatomical facts and injection of local anesthetics despite clear warning signs.

Anesthetists are advised to be aware of the anatomical variations, especially in challenging cases. Misidentification of vertebral level, unrecognized lateral needle placement or deviation, or abnormal caudad termination of the spinal cord can contribute to direct needle injury of the spinal cord [3]. The Tuffer’s line (the line joining the highest level of the iliac crests) is commonly used to identify lumbar interspaces [10]. However, this line does not bear a constant relationship with specific intervertebral levels. Previous studies have shown that Tuffer’s line is unreliable for accurately identifying lumbar intervertebral spaces. Although the cord typically ends opposite the lower border of L1 or the L1-2 interspace, it may extend as low as L3 [11].

Cadaveric studies indicate that the conus medullaris lies below L2 in 43% of women and 27% of men [12,13]. Similarly, an MRI study of 504 adults showed that the spinal cord ends at the L1-2 intervertebral space, reaching below L2 in 20% of the patients. Using the same argument, inserting a needle at the L2-3 level may reach the conus medullaris in approximately 4% to 20% of individuals [14]. Two ultrasound (US) studies further confirmed that the intercristal line is not a reliable indicator of the L4-5 interspace, which is traditionally thought to cross the intercristal line [10,15]. One study found that clinicians’ estimates of the intercristal line often differed from the US measurements. Specifically, clinicians’ estimates were one level higher than the US in 23% of the cases and more than one level higher in 25% of the cases. Agreement with US measurements occurred in only 23% of all assessments [15].

In conclusion, these basic anatomical findings, confirmed using different tools, indicate that the intercristal line determined by palpation is an unreliable anatomical landmark for neuraxial anesthesia [10]. Furthermore, the anesthetists often select a space that is one or more segments higher than the intended level. In such challenging patients, ultrasonography or fluoroscopy should be considered as adjunct tools for accurately determining the vertebral level [3]. 

The second important issue in this case involves the injection of local anesthetic despite severe radiating pain during spinal needle insertion. Radiating pain during needle insertion can indicate intra-neuronal or intra-medullary needle placement [16]. The recommendations of many international guidelines advise that if a patient experiences severe pain during spinal needle insertion, the needle should be withdrawn immediately, and further advancement of the needle or injection of the local anesthetic must be avoided. Furthermore, it is recommended to check the intervertebral space using imaging tools (e.g., US or X-ray), record the incident, and closely monitor the patient to detect early neurological symptoms and signs [3,10,16,17]. Reynolds et al [11] described seven cases of neurological damage following spinal or combined spinal-epidural anesthesia, in which the spinal needle was believed to be introduced at the L2-3 interspace. All patients reported pain during needle insertion, and free-flowing CSF was observed before the spinal injection. Postoperative assessments showed unilateral sensory loss, paresthesia, and pain at the L4-S1 levels, persisted in all patients. Six patients developed foot drop and three experienced urinary symptoms. MRI of the lumbosacral spine showed a spinal cord of normal length with a syrinx in the conus medullaris on the same side as both the persisting clinical deficit and the symptoms that had occurred during needle insertion. In some patients, the conus tip typically lies at the L1-L2 level, although it may extend lower in other patients. These findings emphasize that pain during spinal needle insertion should never be ignored, as it may indicate an abnormally low conus medullaris or needle misplacement. Careful attention to such red flags can prevent severe complications.

Second case

The common peroneal nerve is susceptible to injury along its entire course. The estimated rates of common peroneal nerve palsy seen following total knee arthroplasty TKR range from 0.3% to 4%, while after proximal tibial osteotomy, the rates range from 3% to 13%. The main causes are often surgical, including ischemia, mechanical irritation, traction, crush injury, and laceration, all of which can result in intraoperative nerve injury [18]. Several risk factors have been implicated in the development of peroneal nerve palsy following TKR, including severe valgus deformity, preexisting neuropathy, rheumatoid arthritis, prolonged tourniquet use, external leg compression, lumbar stenosis, and neuraxial analgesia, with the latter representing the lowest incidence among these causes. Most cases of peroneal nerve palsy resolve completely within a few days to weeks [19]. In this case, no deviations from the standard of care were identified by either the anesthetic or surgical teams. Furthermore, the orthopedic consultant clarified from the outset that this complication is a recognized surgical risk and unrelated to the anesthetic technique. Awareness of the causes and the frequency of nerve injuries associated with elective orthopedic surgery can assist anesthesiologists in the diagnosis and treatment of perioperative nerve injuries. Distinguishing between surgical, anesthetic, and patient factors is often challenging [3]. The only concern was the delayed recognition of the complication on the day following surgery. This delay was partly attributed to the primary anesthesia team, who discharged the patient from the PACU before ensuring recovery from spinal or epidural analgesia. Additionally, continuous epidural analgesia was initiated before the patient’s discharge from the PACU, which contradicted the institutional discharge policy and procedure following neuraxial analgesia [20,21].

With the development of newer short-acting local anesthetics, spinal anesthesia has become associated with a rapid onset of action, rapid recovery of motor function, and effective analgesia with minimal side effects compared to general anesthesia [20]. However, Marino et al. [21] found that early PACU discharge did not lead to increased hemodynamic complications in the surgical ward. Thus, discharge from PACU can be performed safely and more efficiently without waiting for the return of motor function in patients receiving spinal anesthesia for THA/TKA, as long as a modified Aldrete score recovery protocol is used.

A multicenter study involving 1,376 patients undergoing lower extremity joint replacement surgery under spinal anesthesia was conducted to assess the benefit of motor assessment before a patient’s discharge from the PACU. The results indicated an insignificant increase in adverse events during the first 24 hours in the ward, although further safety data are required for patients discharged without assessment of lower limb motor function [22]. However, according to our institutional guidelines, no patient should be discharged from PACU unless there is confirmation of at least some motor function recovery following spinal anesthesia (e.g., the ability to move the big toe). The ideal spinal anesthesia would provide rapid onset, optimal surgical conditions, and minimal or no motor blockade at the end of the surgical procedure, thereby enhancing recovery and allowing for early patient discharge [23].

Third case

A report from the ASA Closed Claims study pointed to an increased injury rate in those patients who underwent interventional pain medicine procedures while anesthetized or deeply sedated [24]. Despite the controversy surrounding this topic, the panel advised not to routinely perform regional anesthetic or interventional pain medicine procedures in anesthetized or deeply sedated adult patients [3]. Performing regional anesthesia in deeply sedated or fully anesthetized patients adds an additional risk factor for neuronal damage, as it interferes with the patient assessment regarding paraesthesia, pain during injection, and potential nerve damage. Deep sedation and general anesthesia can also hinder clinical assessment, masking early signs of complications [25]. Evidence from case reports revealed a positive correlation between paresthesia experienced during RA and subsequent nerve injury. Consequently, many sources recommend performing regional anesthesia on awake or lightly sedated patients. However, this recommendation lacks strong scientific evidence indicating that performing RA under general anesthesia or deep sedation poses a greater risk of nerve injury compared to awake or mild sedation [26].

On the other hand, this traditional concept may be attributed to a lack of experienced hands or the limitations of older US machines. Modern high-resolution ultrasound enables precise needle advancement to an extra-epineural position for injection, allowing for injection while avoiding needle-to-nerve contact or intra-epineural injection of local anesthetic. With the use of ultrasound guidance in skilled hands, it is a reasonable option to perform neuraxial and peripheral regional blocks safely in deeply sedated or anesthetized patients. Performing the procedure safely and effectively requires an adequate level of experience with the specific block technique in question [27]. However, recent findings suggest that an anesthetist’s experience, education level, and training play an important role in performing RA, especially in specific situations such as with sedated and anesthetized patients. The incidence of incorrect needle positioning, resulting in intraneural injection, appears to be higher than previously anticipated [28]. Intraneural injection itself may not necessarily be the direct cause of nerve injury; instead, inflammatory responses secondary to nerve irritation may potentially contribute to the observed perioperative nerve injury [1]. This may also explain why partial or complete recovery of the injured nerves is often observed within days or even weeks. Paresthesia or pain on injection in a responsive patient is an important warning sign. Consensus guidelines recommend that nerve blocks in adults be undertaken in an awake, responsive patient, which allows for symptoms of local anesthetic systemic toxicity (LAST) to be reported. However, exceptions are adults at risk of movement during the block procedure and children, where nerve blocks may be performed under anesthesia [3,29].

Fourth case

Spinal epidural lipomatosis (SEL) is a rare disease characterized by an abnormal accumulation of adipose tissues in the epidural space, leading to spinal canal stenosis with subsequent compression of the neural structures [30]. The incidence of SEL has been reported up to 6.26% of patients presenting with spinal stenosis [31]. This case represents a unique scenario involving a patient with spinal epidural lipomatosis (SEL) that leads to motor weakness after an epidural steroid injection. The neurological deficits were mainly attributed to inadequate patient assessment, missed documentation review, previous adverse effects following L-ESI, patient non-compliance, and the patient’s denial of any prior symptoms. An MRI conducted two years prior showed no evidence of SEL, whereas a recent MRI following the current neurological incident indicated clear evidence for the presence of SEL. The symptoms of SEL vary and range from back pain, myelopathy, radiculopathy, neurogenic claudication, loss of sensation, lower extremity weakness, and, in rare cases, paraplegia. In severe cases, treatment options include endoscopic and minimally invasive intervention [31] or even decompressive laminectomy is necessary when required [32].

The exact etiology of SEL remains unclear. However, SEL is strongly associated with steroid use and obesity. Studies have shown a positive correlation between the frequency of steroid injections and the incidence of SEL [33]. This correlation aligns with the present case scenario, where the patient had no neurological deficits following the first L-ESI. However, the second L-ESI was followed by transient motor weakness that resolved within one week, while the third L-ESI, administered at our hospital, resulted in more severe neurological symptoms that persisted for several weeks. 

There was some controversy regarding the use of L-ESI in patients with symptomatic SEL. Severe spinal stenosis, and specific space-occupying extradural lesions (eg, epidural lipomatosis, ligamentum flavum hypertrophy, synovial cysts, or ependymoma) have been associated with temporary or permanent spinal cord injury in conjunction with neuraxial regional anesthetic techniques [3]. Typically, a single dose of ESI is not enough to stimulate epidural fat accumulation [34]. Consequently, the first epidural analgesia can be performed with the use of steroids. However, subsequent epidurals particularly in the short term, should be administered using only local anesthetics to minimize the risk of exacerbating SEL [30].

Proper documentation review and repeated clinical assessments are essential to identify and eliminate any risk factors and ensure patient safety before conducting interventional procedures [35]. Meanwhile, inadequate pre-procedure assessment and failure to review previous patient records may lead to complications. In recent years, there has been an increase in the use of invasive procedures. Concomitantly, there has also been an increase in the complications associated with these procedures. Taking this into consideration, healthcare providers need to adopt a cautious and vigilant approach, with a focus on patient safety, aiming to minimize the risk of adverse events by careful patient selection, the use of proper techniques, close post-procedure monitoring, and follow-up [36]. However, the non-compliant behavior of patients and denial of prior adverse events frequently interferes with the effectiveness of treatments across various medical conditions and can lead to serious consequences [37]. Clinicians are well aware that patients may deny a variety of clinical realities or unfavorable side effects. The underlying reasons for this denial are not entirely clear; it may be related to psychological defense mechanisms, perceived rewards, or a lack of understanding regarding the impacts of this behavior on the treatment outcome. Denial can result in treatment delays and may jeopardize patient safety [38]. In this case, the patient’s explanation later on after the event was mainly related to failure to understand the relation between the previous incident and the outcome of the proposed pain intervention. She thought that the previous incident was related to the level of care and experience between the staff at the two hospitals.

Fifth case

Patients with preexisting musculoskeletal deformities, combined with suboptimal positioning during prolonged orthopedic surgery under general anesthesia, are recognized risk factors for postoperative neurological deficits. In addition, obtaining written consent, rather than relying solely on oral consent, along with conducting in-depth discussions with the patient about the risks and benefits is essential before any intervention. Ideally, patients should be fully informed and allowed to make independent decisions without undue persuasion [4]. These factors collectively increase the risk of neurological injury and are likely to contribute to adverse outcomes following neuraxial analgesia [3].

The presence of musculoskeletal deformities may affect patient positioning during surgery, hinder access to regional anesthesia, and obscure anatomical landmarks. Improper positioning of the patient on the operating table can result in regions of the body without adequate support requiring additional care during anesthesia [39]. The patient had a long history of rheumatoid arthritis which is characterized by destruction of synovial joints, primarily affecting the small joints, as well as the spine joints which are of particular concern to anesthesiologists. Furthermore, orthopedic surgeries are among the most common procedures performed in patients with RA, and most regional anesthetic methods may be applied. RA in patients with deformities may present challenges for regional anesthesia due to difficulties in positioning during surgery and limited access to regional techniques. However, the most common contraindications for the use of regional techniques are patient refusal, use of anticoagulant therapy, infection at the puncture site, and hemodynamic instability [40]. An additional risk for neurologic complications may also result from the required positioning during surgery. Mechanisms of nerve injury related to surgery include traction, transection, compression, contusion, ischemia, and stretch [17]. During surgery, patients are placed in positions they would not otherwise tolerate unless anesthetized. In addition, the physical forces required during surgery (e.g., placement of prostheses) can be excessive, potentially stressing anatomical structures remote from the surgical site, including the vertebral column [3,17]. Awareness of suboptimal surgical positioning should prompt consideration of risk-vs-benefit when contemplating epidural blocks under GA [3]. This topic remains controversial due to concerns about the inability of anesthetized patients to respond to pain and potentially increasing the risk of neurologic complications [41]. Paresthesia and pain during nerve block placement and LA injection have been recognized as risk factors for serious neurologic deficits following regional techniques. As a result, many experts emphasize the importance of close communication with the patient during neuraxial procedures as a critical component of safe practice. Current evidence supports performing epidural insertion in awake or minimally sedated patients to ensure safety [17,27]. However, needle and catheter placement in anesthetized adults may be an acceptable alternative in carefully selected cases. Studies of lumbar epidural insertion during GA have reported a low risk of neurologic complications [17,27,41]. Despite these findings, the relative risk of epidural block administration in anesthetized patients versus awake patients remains uncertain (Table 1).

**Table 1:** Deviations from the standard of care and lessons learned from each case PACU: post-anesthesia care unit, ESI: epidural steroid injection

Case report	Case-specific deviations from the standard of care	Lessons learned for improvements
First case [3,10,22]	Notable deviations included selecting a higher intervertebral level for spinal anesthesia based on the palpation of the intercristal line (Tuffer’s line), which ultimately led to spinal cord injury at the cons medullaris. Additionally, an inappropriate intrathecal injection was performed despite clear warning signs (severe, shooting, electric-like pain radiating to the lower limb), and there was a lack of immediate follow-up by the primary anesthesia team, which delayed the recognition of complications.	This case emphasizes the importance of considering the basic anatomical knowledge, using imaging tools (such as ultrasound or fluoroscopy) to determine the correct intervertebral level, especially in obese patients. Avoiding intrathecal injection when warning signs like severe pain are present. Immediate postoperative follow-up is essential, especially challenging and difficult cases.
Second case [21,22]	There were no actual deviations from the standard of care either by the anesthesia or orthopedic teams. However, a concern was noted regarding the patient discharged from the PACU before confirming the recovery from spinal anesthesia as recommended by the guidelines. This case highlights effective communication among the different management teams.	It is essential to ensure that no patient is discharged from the PACU without confirmation of motor function recovery following spinal anesthesia.
Third case [18,27]	The peripheral nerve block was performed by an inexperienced resident while the patient was under general anesthesia which likely led to an increased risk of intraneural injection.	Ultrasound-guided peripheral nerve blocks should not be performed while the patient is deeply sedated or under general anesthesia. In such cases, the procedure should be conducted only by a senior and experienced anesthesiologist.
Fourth case [3,4]	This case differs from previous cases in that there were lapses by both the team and the patient as well. The deviation from the standard of care is a double face by the team and by the patient as well. The team conducted insufficient history-taking and did not perform a detailed review of the patient’s prior medical records. Additionally, the Patient denied any prior procedures or adverse events following previous ESI.	This case underscores the critical importance of thoroughly history-taking and a detailed review of all patient documentations to ensure comprehensive and accurate care. Involving patients in the treatment decisions helps them better understand their condition, supports shared decision-making, and encourages them to recognize the impact of their choices.
Fifth case [4,5,37]	The anesthesia team attempted to convince the patient to accept the epidural analgesia and no written informed consent was taken for epidural anesthesia. Additionally, there were other risk factors in such patient: she has musculoskeletal deformities, was scheduled for major orthopedic procedure and in non-optimal position. The epidural anesthesia was performed while the patient under general anesthesia. Moreover, the continuous epidural analgesia initiated in the PACU before patient’s recovery from neuraxial anesthesia. The patient also appeared to have an unstable personality, was noncompliant with treatment plan.	The main role of the anesthesia team is to provide a clear explanation of the risks and benefits of any regional technique such as the epidural analgesia, without pressuring the patient to accept it. A written informed consent is standard practice, rather than relying on verbal consent. A preoperative psychiatry consultation could have been beneficial to help stabilize the patient during the perioperative period. Therefore, it is recommended to avoid regional anesthesia in patients with multiple risk factors for neurological complications after surgery.

Limitations

The manuscript has several limitations that warrant consideration. First, its retrospective design may introduce selection bias and lead to incomplete data collection. In addition, the analysis is based on small sample size, focusing on only five cases, which limits the generalizability of the findings to broader populations. Lastly, the follow-up period of three to six months is relatively short, leaving the long-term neurological outcomes and recovery course of some patients inadequately explored.

Recommendations

In the event of a neurological complication following regional anesthesia and pain interventions, consider the following actions as early as possible to ensure rapid recovery and patient safety:

Obtain a Second-Party Consultation

A neurologist can provide an unbiased evaluation, reducing potential tension or anxiety for both the patient and the primary provider [1,3].

Prompt Imaging for Neuraxial Anesthesia Concerns

If there is any suspicion of neurological deficits following regional techniques or pain procedures, emergent imaging such as X-ray, MRI, or CT should be performed without delay [14,15,42].

Early Neurophysiological Studies

These studies help differentiate between preexisting versus potentially recent injuries, determining the level of neuronal injury, and differentiating between central and peripheral lesions (e.g., neuropathy or plexopathy). Moreover, neurophysiological studies are also valuable for patient follow-up [14,15,42].

Tuffier's Line

The intercristal line is an unreliable method for identifying the lumbar interspaces. Anesthetists should be cautious not to insert a spinal needle above the “L3” level, especially in women during cesarean section [10,11].

Involving the Patient in the Management Decision

Engaging the patient in the treatment options can enhance their understanding and shared decision-making approach and responsibility, ensuring that they are fully informed and aware of the implications of their choices [4,37,38].

Informed Written Consent

Valid informed consent requires patients to be fully aware of treatment risks, alternatives, and benefits, with decisions made voluntarily and without pressure. For consent to be meaningful, four conditions must be met: voluntariness, disclosure of risks, patient understanding of the treatment and its outcomes, and the capacity to make autonomous decisions aligned with their goals [4].

Adopt Multidisciplinary Team Management

These cases highlight the importance of a collaborative, multidisciplinary approach when treating patients with neurological and psychological symptoms following regional techniques or pain interventions [3,42].

Role of Training

Effective training plays a vital role in minimizing neurological complications after regional anesthesia by enhancing practitioner’s skills and knowledge [3,17].

Comprehensive Training Programs

They ensure that anesthetists have a thorough understanding of anatomy and physiology, enabling precise needle placement, and reducing the risk of inadvertent nerve injury [3,17].

Professionalism in Using Imaging Tools

Proficiency in ultrasound and fluoroscopy guidance is crucial, as it provides real-time visualization, helps to identify anatomical variations, avoids important structures, and reduces the risk of neurological complications [3,17].

## Conclusions

A thorough understanding of the risk factors and pathophysiology underlying neuraxial and peripheral nerve injuries is essential for reducing the incidence of adverse neurological outcomes. Actively involving patients in the decision-making process enhances their understanding, promotes a sense of responsibility, and ensures they are fully aware of the implications of their choices. Furthermore, effective training and continuous education are vital for improving practitioner’s skills and knowledge, thereby minimizing neurological complications associated with regional anesthesia. Anesthesiologists and pain specialists must be familiar with the evidence-based guidelines and ensure their strict adherence at every step of the procedure, to enhance the quality of care and ensure patient safety.
